# Systematic Study on Genetic and Epimutational Profile of a Cohort of Amsterdam Criteria-Defined Lynch Syndrome in Singapore

**DOI:** 10.1371/journal.pone.0094170

**Published:** 2014-04-07

**Authors:** Yanqun Liu, Min Hoe Chew, Xue Wei Goh, Soo Yong Tan, Carol Tien Tau Loi, Yuen Ming Tan, Hai Yang Law, Poh Koon Koh, Choong Leong Tang

**Affiliations:** 1 Department of Colorectal surgery, Singapore General Hospital, Singapore, Singapore; 2 Department. of Pathology, Singapore General Hospital, Singapore, Singapore; 3 Department of Paediatric Medicine, KK Women's and Children's Hospital, Singapore, Singapore; Sapporo Medical University, Japan

## Abstract

**Background:**

Germline defects of mismatch repair (MMR) genes underlie Lynch Syndrome (LS). We aimed to gain comprehensive genetic and epigenetic profiles of LS families in Singapore, which will facilitate efficient molecular diagnosis of LS in Singapore and the region.

**Methods:**

Fifty nine unrelated families were studied. Mutations in exons, splice-site junctions and promoters of five MMR genes were scanned by high resolution melting assay followed by DNA sequencing, large fragment deletions/duplications and promoter methylation in *MLH1*, *MSH2*, *MSH6* and *PMS2* were evaluated by multiplex ligation-dependent probe amplification. Tumor microsatellite instability (MSI) was assessed with five mononucleotide markers and immunohistochemical staining (IHC) was also performed.

**Results:**

Pathogenic defects, all confined to *MLH1* and *MSH2*, were identified in 17 out of 59 (28.8%) families. The mutational spectrum was highly heterogeneous and 28 novel variants were identified. One recurrent mutation in *MLH1* (c.793C>T) was also observed. 92.9% sensitivity for indication of germline mutations conferred by IHC surpassed 64.3% sensitivity by MSI. Furthermore, 15.6% patients with MSS tumors harbored pathogenic mutations.

**Conclusions:**

Among major ethnic groups in Singapore, all pathogenic germline defects were confined to *MLH1* and *MSH2*. Caution should be applied when the Amsterdam criteria and consensus microsatellite marker panel recommended in the revised Bethesda guidelines are applied to the local context. We recommend a screening strategy for the local LS by starting with tumor IHC and the hotspot mutation testing at *MLH1* c.793C>T followed by comprehensive mutation scanning in *MLH1* and *MSH2* prior to proceeding to other MMR genes.

## Introduction

Lynch Syndrome (LS), (previously referred to as hereditary nonpolyposis colorectal cancer, HNPCC), is an autosomal, dominantly inherited syndrome associated with substantial risks for colorectal cancers (CRC). It is also associated with increased malignant risk of the endometrium, stomach, ovaries, small bowel, ureter, billary tract, renal pelvis, brain (Turcot syndrome) and skin (Mure Torre syndrome) [1]. Inactivating mutations in at least four mismatch repair (MMR) genes (*MLH1*, *MSH2*, *MSH6* and *PMS2*) have been identified [2] and account for 50–60% of cases that fulfill clinical criteria [3]–[5]. Mutations are most commonly identified in *MLH1* (50%) and *MSH2* (40%). 7% of the mutations are also found in *MSH6* and 3% in PMS2 [2]. Genetic defects in LS are however highly heterogeneous. Over 600 causative mutations have been reported scattered throughout MMR genes with no obvious mutation hotspots (Atlas of Genetics and Cytogenetics in Oncology and Haematology, http://AtlasGeneticsOncology.org). The majority of these descriptions arise from Western populations. Large genomic rearrangement (one or multi-exonic deletions or duplications) in MMR genes [6]–[8] account for variable proportions of inherited defects in Western populations. Constitutional epimutation (promoter methylation) resulting in transcriptional silencing of MMR genes have also been attributed as the cause of disabled mismatch repair functions in some LS cases [9], [10]. Interestingly, large deletions of the 3′ end of the *EPCAM* gene would lead to promoter methylation of the downstream *MSH2*, causing *MSH2*-associated LS [11], [12].

Clinical guidelines for LS were developed to facilitate linkage and positional cloning studies. Amsterdam I and II criteria were highly specific to reduce misclassification of families but suffered from a lower sensitivity. Japanese criteria were introduced with slightly less stringent conditions. Revised Bethesda guidelines including pathological features were subsequently created as an even more widely embracing approach for diagnosis of the syndrome (Table S1) [13]–[16]. In Western populations, traditional description of HNPCC-associated CRC was predominantly right sided. In contrast, several Asian reports including one from our unit have suggested a left sided predominance [17], [18]. The observed variance in phenotypic manifestation may reflect true ethnic or geographic variations in LS phenotypic expression and underlying genomic defects. These also suggest that the Amsterdam criteria may be inadequate for clinical diagnosis of LS when applied to Asian populations.

It is increasingly recognized that there is a variety of mutational spectrums and frequencies of major MMR genes in different geographic regions and ethnic groups [19]. This understanding is critical to the development of efficient molecular diagnostic strategies in each population. In our country, there are three dominant ethnic groups that reflect the South-East Asian region: namely Chinese, Malays and Indians. To date, except for one report defining *MLH1* and *MSH2* mutations in five Singaporean Amsterdam-defined LS families [20], there is no comprehensive MMR gene mutational profile available for South-East Asian clinically-diagnosed LS kindreds. In this present study, we aim to systematically study the molecular characteristics of five MMR genes (*MLH1*, *MSH2*, *MSH6*, *PMS2* and *PMS1*) in an Amsterdam-defined cohort in our local population.

## Methods

### Ethnic statement

This study was approved by the Institutional Review Board of the Singapore General Hospital, Singapore. Study participants gave written informed consent after verbal counseling according to the protocols approved by the Board.

### Subjects/Patients

A total of 91 subjects (71 cancer, seven adenoma patients and 13 unaffected family members) from 59 unrelated families from Singapore Polyposis Registry, were selected for this study. Patients fulfilled either the Amsterdam I (AC-I, 29 families) and/or II criteria (AC-II, 28 families) (Table S1). In addition, we also included two families that fulfilled Japanese criteria due to small family size (Table S1). In these two families, there are two CRCs in first-degree relatives involving two consecutive generations and age of CRC onset in one patient in each family was under 30 years. Details on the Singapore Polyposis Registry, collection of clinoco-pathological data and generation of pedigrees of LS patients have been previously described [18].

### Study design

**Figure 1:**
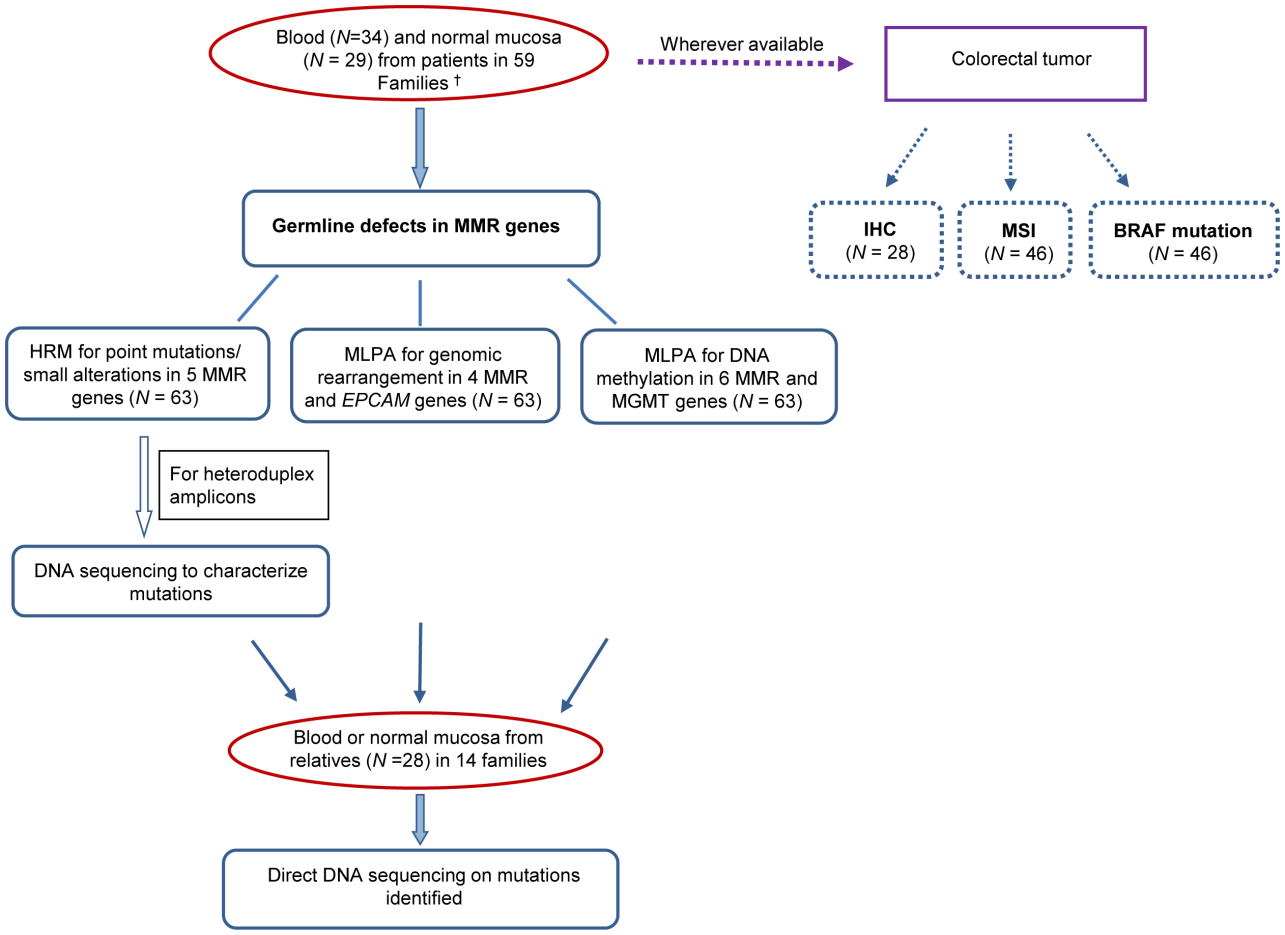
Flow diagram of the analytical strategy. Fifty nine unrelated families were screened for genetic defects in common MMR genes with all tests performed in parallel without using any sets of results for subsequent assays. Germline gene status was investigated by utilizing DNA extracted from blood or normal mucosa. Additional analyses in tumor tissues, such as immunohistochemical (IHC) assessment, microsatellite instability (MSI) test and mutation detection of *BRAF* V600E were performed where tumor materials were available. When a pathogenic or suspected deleterious defect was identified, family relatives were screened for presence of the same defect. ^†^, at least one CRC patient each from 59 unrelated families.

Colorectal tissues (tumor and adjacent normal mucosa, at least 5 cm away from tumor lesions) were collected from enrolled patients, and blood from probands and relatives. All molecular assays were performed in parallel without using any sets of results for subsequent assays (Figure 1). Germline DNA from 63 CRC patients (at least one patient each from 59 unrelated families) was subject to high resolution melting (HRM) procedure followed by DNA sequencing, and multiplex ligation-dependent probe amplification (MLPA). Additional investigations in tumor tissues, such as immunohistochemical (IHC) assessment of MMR proteins, microsatellite instability (MSI) test and mutation detection on *BRAF* V600E were performed where tumor materials were available. When a pathogenic or suspected deleterious defect was identified, family relatives were screened for presence of the same defect. In total, 91 DNA samples were examined. The detailed description of each assay is provided below.

### Germline defect detection in MMR genes

Genomic DNA was extracted using standard phenol/chloroform/isoamyl alcohol method. DNA extracted from leucocytes and/or normal colorectal mucosa in 63 CRC patients was subjected to PCR amplification with amplicons covering all exons, exon-intron junctions and promoters of all five genes (*MLH1*, *MSH2*, *MSH6*, *PMS2* and *PMS1*). The amplicons were then subjected to mutation scanning via the HRM procedure in the LightScanner System (Idaho Technology Inc., Salt City, USA) with heteroduplexes pinpointed thereafter being sequenced to characterize the mutations by using BigDye Terminator v3.1 kit on the ABI PRISM 3100 Genetic Analyzer (Applied Biosystems Inc., Foster City, USA). Mutations, mainly point mutations, small deletions/insertions, or splice site alterations, were confirmed by re-sequencing in both directions. With that, genomic DNA from other family members was subjected to direct sequencing targeted at the mutations identified in the index cases of the families.

Large genomic rearrangement in all exons of *MLH1*, *MSH2*, *MSH6* and *PMS2* as well as exons 3, 8 and 9 of *EPCAM* was examined by MLPA assay according to manufacturer's protocols (SALSA P003 and P248 for *MLH1*/*MSH2*, P008 for *PMS2*, and P072 for *MSH6* and *EPCAM*, from MRC-Holland, Amsterdam, The Netherlands). The exon deletion was defined if the relative peak area was reduced by 35–55%, and exon duplication as increase by 35–55%.

Promoter methylation of *MLH1*, *MSH2*, *MSH6* and *PMS2*, and other three genes (*MLH3*, *MSH3* and *MGMT*) was also investigated and analysed by using a methylation-specific MLPA kit according to manufacturer's instructions (MRC-Holland, Amsterdam, the Netherlands). A dosage ratio of 0.15 or higher, corresponding to 15% of methylated DNA, was interpreted to indicate promoter methylation based on a previous study [9].

### Definition of pathogenic mutations

Sequencing variants such as nonsense mutations, frameshift mutations, and large genomic deletions of one or more exons were considered pathogenic. Aberrant methylation (≥15% methylation ratio) at regions influencing promoter activity was also considered pathogenic. All missense mutations were checked against two well-established and most relevant databases, the LOVD database maintained by International Society for Gastrointestinal Hereditary Tumours (InSiGHT, www.insight-group.org) and the Human Gene Mutation Database (www.hgmd.cf.ac.uk/ac/index.php) to determine their pathogenicity. A missense variant was considered pathogenic only if it was classified as pathogenic or disease causing by both databases. The remaining non-synonymous missense mutations, in-frame deletions, nucleotide substitution in introns or promoters with biological significance to be elucidated were categorized as variants of uncertain significance (VUS).

### Tumor MSI analysis

Tumor tissues were micro-dissected via the standard protocol as described previously [21]. Cancerous cells accounted for at least 85% of the total cells harvested. Isolated DNA from processed tumor and adjacent normal mucosal tissues was tested with a panel of microsatellite markers recommended in the revised Bethesda guidelines-BAT-25, BAT-26, NR-21, NR-24, MONO-27. A commercial MSI analysis kit was used according to manufacturer's protocol (Promega, Madison, WI). Additional peaks at microsatellite loci in the carcinoma compared with matched normal tissue were interpreted as microsatellite instability. MSI-H was defined as instability shown in two or more markers, MSI-L in only one marker, while MSS in none of the five markers.

### Tumor *BRAF* gene mutation detection

To differentiate MSI-H tumors associated with LS from those with non-hereditary origin, the mutation hotspot (c.1779T>A, p.V600E) of *BRAF* was scanned via HRM followed by direct sequencing with Big Dye terminator cycling sequencing kit again.

### Tumor immunohistochemical staining

IHC for MLH1, MSH2, MSH6 and PMS2 proteins was performed on formalin-fixed paraffin embedded tissue sections by a horseradish peroxidase-labelled, polymer-based technique on the Bondmax automated stainer according to manufacturer's protocols (Leica Biosystems, Wetzlar, Germany). Mouse monoclonal antibodies used were: anti-MLH1 (clone No. G168-728 from BD Biosciences, San Diego, CA, diluted 1∶100); anti-MSH2 (Clone No. FE11 from Biocare Medical, Concord, CA, diluted 1∶50); anti-MSH6 (Clone No. BC/44 from Biocare Medical, Concord, CA, diluted 1∶50); anti-PMS2 (clone M0R4G, from Leica, Newcastle, UK, diluted 1∶50). Slides were counterstained with hematoxylin.

**Figure 2:**
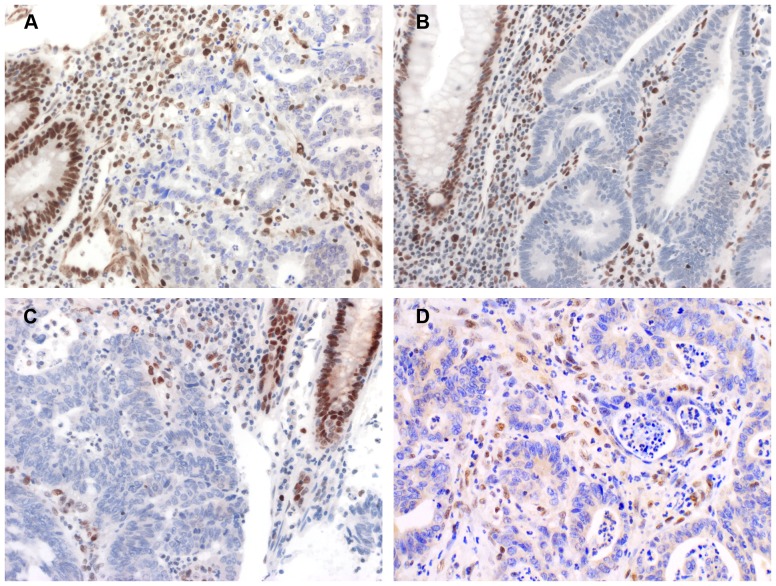
Examples of immunostains showing loss of MMR proteins. Positive nuclear staining in normal colonic epithelium or stromal cells and loss of expression in colorectal cancer of MLH1 (A), MSH2 (B), MSH6 (C) and PMS2 (D).

All IHC results were reviewed and evaluated independently by a dedicated pathologist and an experienced scientist blind to the molecular results. Normal expression of protein was defined as the presence of nuclear staining in colorectal cancer cells. Negative nuclear staining in neoplastic cells with concurrent positive nuclear staining in normal colonic crypt epithelium adjacent to the tumor, lymphoid or stromal cells indicated loss of protein expression. Tumors were scored “equivocal” if nuclear staining was weak or positive nuclear staining was present in <10% neoplastic cells, Representative images showing loss of MMR protein expression in CRC tumors are displayed in Figure 2.

### Statistical analysis

T-test followed by Bonferroni correction was employed to compare the differences of age at onset of cancers. Categorical variables were compared with either the chi-square test (Somers' D test) or Fisher's exact test where appropriate. Statistical analysis was performed using the Statistical Package for Social Sciences version 17.0 (SPSS, Chicago, Illinois). All statistical tests were two-sided and *P* values less than 0.05 were considered statistically significant.

## Results

### Clinical characteristics

In this study cohort, the majority were Chinese families (*N* = 52, 52/59  = 88.1%), followed by six Malay (10.2%) and one Eurasian family (1.7%). Out of 78 patients, 40 were defined by AC-I, 36 by AC-II, and 2 by Japanese criteria. There were 39 patients in each gender group. Median age of initial cancer diagnosis was 44 years (range: 22-84), while the median age specific for CRC diagnosis was 45 years. The majority of CRC were left-sided (44 out 65 CRC, 67.7%). The incidence of synchronous CRC was 4.6% (*N* =  3/65), and metachronous CRC lesions were 6.2% (*N* =  4/65). The most common extra-colonic malignancy was endometrial cancer (5/8 =  62.5%), followed by ovarian cancer (2/8 = 25.0%).

### Mutational and epimutational spectrum in 59 LS families

A total of 60 genetic variants were identified in 40 out of 59 families. These include point mutations, small insertions, small deletions, indels, and a large deletion. Among these variants, 15 were pathogenic and occurred in 17 families, demonstrating the LS rate of 28.8% (17/59) in this cohort. These pathogenic variants were found in *MLH1* (9 variants in 11 families) and *MSH2* (6 variants in 6 families). It was noted that a single distinct pathogenic mutation was usually found in each family. Interestingly, one pathogenic point mutation in *MLH1* (c.793C >T) leading to skipping of exon 10 occurred in three unrelated Chinese families (Table 1). A large deletion spanning exons 1–6 of *MSH2* was identified in a Chinese family in which expression of MSH2 protein was lost in proband's colon tumor. Except that modest (9–10%) methylation was noted at promoter (c.-659 to c.-246) of *MLH1* in one patient, no other methylation was observed in this series.

**Table 1:** Pathogenic mutations identified in 17 Amsterdam-criteria-defined families in Singapore.

Gene/location	Nucleotide change	Protein effect	Ethnicity	Family (*N*)	Patient (*N*)	LOVD †	HGMD ‡	Have been reported?
*MLH1*/Exon 1	c.104-105insAA	p.Met35IlefsX6	Chinese	1	1	Pathogenic	Dis causing	Yes
*MLH1*/Exon 6	c.462delC	**p.Asp154AspfsX5**	Chinese	1	1	No record	No record	**Novel pathogenic**
*MLH1*/Exon 8	c.604delG	**p.Ala202LeufsX27**	Chinese	1	2	No record	No record	**Novel pathogenic**
*MLH1*/Intron 9	c.790+1G>A	Splicing change, skipping of exons 9 and 10	Chinese	1	1	Pathogenic	Dis causing, exon skipping	Yes, reported 37 times
*MLH1*/Exon 10	c.793C>T	Splicing change, skipping of exon 10	Chinese	3	5	Pathogenic	Dis causing, exon skipping	Yes, founder mutation in Taiwanese Chinese [22]
*MLH1*/Exon 14	c.1636-1640insAAGTT	**p.Lys546LysfsX46**	Chinese	1	3	No record	No record	**Novel pathogenic**
*MLH1*/Exon 14	c.1657delACCAAGCTTAG	**p.Thr553X**	Malay	1	1	No record	No record	**Novel pathogenic**
*MLH1*/Exon 17	c.1944delCCinsG	**p.Pro648PrpfsX12**	Chinese	1	1	No record	No record	**Novel pathogenic**
*MLH1*/Exon 18	c.2041G>A	p.Ala681Thr	Chinese	1	1	Pathogenic	Dis causing	Yes, reported 68 times
*MSH2*/Exons 1–6	Deletion of exons 1–6	Deletion of exons 1–6	Chinese	1	1	Pathogenic	Dis causing	Yes, founder mutation in North America [24]
*MSH2*/Exon 3	c.508C>T	p.Gln170X	Chinese	1	1	Pathogenic	Dis causing	Yes, reported 4 times
*MSH2*/Exon 7	c.1216C>T	p. Arg406X	Malay	1	2	Pathogenic	Dis causing	Yes, reported 36 times
*MSH2*/Exon 9	c.1457–1460del	p. Asn486ThrfsX10	Chinese	1	1	Pathogenic	Dis causing	Yes, founder mutation in Hong Kong [34]
*MSH2*/Exon 13	c.2041C>T	**p.Gln681X**	Eurasian	1	1	No record	No record	**Novel pathogenic**
*MSH2*/Intron 13	c.2210+1G>A	Skipping of exon 13	Chinese	1	1	Likely pathogenic	Dis causing	Yes, reported twice

† , LOVD, London open variation database (V2.0 build 35) maintained by International Society of Gastroenterological Hereditary Tumors (InSiGHT). Variants were classified according to Consensus InSiGHT Classification v.1.9: 5/09/2013.

‡ , HGMD, human gene mutation database. Dis causing, disease causative mutation; Our data were checked against this database as of 10 Jan 2014.

Putative novel mutations were highlighted in bold.

Interestingly out of the 15 pathogenic mutations identified, six were novel variants (five in *MLH1* and one in *MSH2*) that have never been previously described in common MMR databases (Table 1). Out of 28 VUS identified (Table S2), 20 were novel [three VUS in *PMS2*, four each in *MLH1*, *MSH2* and *MSH6,* and five in *PMS1*]. In addition, we have found 17 single nucleotide polymorphisms (SNPs) and some of which are reported to be associated with increased risks of LS (Table S2). Among these SNPs, the two located at intron 9 of *PMS2* were novel SNPs as polymorphisms were newly observed in our series.

### Concordance between tumor IHC stains and germline mutations

In this cohort with 65 CRC patients, we have 28 samples where correlation was assessable between germline mutations and tumor IHC staining. MMR protein expression in tumors was in concordance with germline status of respective genes in 21 patients. Non-concordance was observed in two patients, while other five cases were inconclusive as biological impact of the variants identified needs to be characterized in the future (Table 2). As such, in 23 patients with interpretable results, the concordance rate between tumor IHC and germline status of the MMR genes was 91.3% (21 out of 23). Further correlation analysis with pathogenic mutations identified demonstrated that 13 out of 14 colorectal tumors exhibited negative staining of respective MMR proteins. The sensitivity of IHC being indicative of germline defects is thus 92.9%.

**Table 2:** Correlation between tumor immunohistochemical (IHC) stains and germline mutations in 28 suspected Lynch Syndrome patients.

			IHC					
DNA ID	Nucleotide change and effect on protein	Nature of mutation	MLH1	MSH2	MSH6	PMS2	IHC – Mutation concordance	IHC Sensitivity $
60	*MLH1*/Exon1, c.104-105insAA, p. Met35IlefsX6	Pathogenic	Lost	Normal	Normal	Lost	Yes	Yes
45	***MLH1*** **/Exon 8, c.604delG, p.Ala202LeufsX27**	**Novel Pathogenic**	Lost	Normal	Normal	Lost	Yes	Yes
39	***MLH1*** **/Exon 8, c.604delG, p.Ala202LeufsX27**	**Novel Pathogenic**	Lost	Normal	Normal	Lost	Yes	Yes
51	*MLH1*/Intron 9, c.790+1G>A	Pathogenic	Lost	Normal	Normal	Lost	Yes	Yes
32	*MLH1*/Exon 10, c.793C>T, p.Arg265Cys	Pathogenic	Lost	Normal	Normal	Lost	Yes	Yes
36	***MLH1*** **/Exon 14, c.1636-1640insAAGTT, p.Lys546LysfsX46**	**Novel Pathogenic**	Lost	Normal	Lost	Lost	Yes	Yes
26	***MLH1*** **/Exon 14, c.1636-1640insAAGTT, p.Lys546LysfsX46**	**Novel Pathogenic**	Lost	Normal	Normal	Lost	Yes	Yes
136	***MLH1*** **/Exon 14, c.1657delACCAAGCTTAG, p.Thr553X**	**Novel Pathogenic**	Lost	Normal	Normal	Lost	Yes	Yes
29	***MLH1*** **/Exon 17,c.1944delCCinsG, p.Pro648PrpfsX12**	**Novel Pathogenic**	Lost	Normal	Normal	Lost	Yes	Yes
22	*MSH2* deletion of exons 1-6	Pathogenic	Normal	Lost	Lost	Normal	Yes	Yes
21	*MSH2*/Exon 3, c.508C>T, p.Gln170X	Pathogenic	Normal	Lost	Equivocal	Equivocal	Yes	Yes
52	***MSH2*** **/Exon 13, c.2041C>T, p.Gln681X**	**Novel Pathogenic**	Normal	Lost	Lost	Normal	Yes	Yes
27	*MSH2*/Intron 13, c.2210+1G>A	Pathogenic, skipping of exon	Normal	Lost	Lost	Normal	Yes	Yes
7	No mutation detected	No mutation detected	Normal	Normal	Normal	Normal	Yes	
422	No mutation detected	No mutation detected	Normal	Normal	Normal	Normal	Yes	
468	No mutation detected	No mutation detected	Normal	Normal	Normal	Normal	Yes	
462	No mutation detected	No mutation detected	Normal	Normal	Normal	Normal	Yes	
495	No mutation detected	No mutation detected	Normal	Normal	Normal	Normal	Yes	
43	No mutation detected	No mutation detected	Normal	Normal	Normal	Normal	Yes	
393	*MLH1*/Exon 18, c.2041G>A, p.Ala681Thr	Pathogenic	Normal	Normal	Normal	Lost	No	Missed
157	*MLH1*, promoter methylation	Uncertain impact on gene silencing due to modest (9–10%) methylation	Lost	Normal	Normal	Lost	Yes	NA due to uncertainty
81	*MLH1*/Exon 18, c.2042 C>T, p.Ala681Val	Discrepancy by InSiGHT † and HGMD ‡ classification criteria	Lost	Normal	Normal	Lost	Yes	NA due to Discrepancy in two databases.
448	***MLH1*** **/Exon 1, c.101**–**102delAGinsTT, p.Glu34Val**	**Inframe indel, novel VUS**	Normal	Normal	Normal	Lost	No	NA
368	*MLH1*/Intron 13, c.1558+14G>A; ***PMS2*** **/Intron 11, c.2006 +6 G>A**	*MLH1*, probably dis causing by HGMD criteria;‡ ***PMS2*** **, Novel VUS**	Normal	Normal	Normal	Normal	Inconclusive due to uncertain impact	
68	*MSH2*/Exon 7, c.1168C>T, p.Leu390Phe	Probably dis causing by HGMD criteria ‡	Normal	Normal	Normal	Normal	Inconclusive due to VUS	
8	***MSH6*** **/Exon 4, c.2246G>C, p.Gly749Ala**	**Novel VUS**	Normal	Normal	Normal	Normal	Inconclusive due to VUS	
409	***PMS2*** **/Exon 7, C.718 A>T, p.Ile240Phe**	**Novel VUS**	Normal	Normal	Normal	Normal	Inconclusive due to VUS	
438	*PMS2*/Intron 11, c. 2006+6G>A	VUS	Normal	Normal	Normal	Normal	Inconclusive due to VUS	

$ , Tumor IHC sensitivity for germline defects.

† , LOVD, London open variation database (V2.0 build 35) maintained by International Society of Gastroenterological Hereditary Tumors (InSiGHT). Variants were classified according to Consensus InSiGHT Classification v.1.9: 5/09/2013.

‡ , HGMD, human gene mutation database. Dis causing, disease causative mutation; Our data were checked against this database as of 10 Jan 2014.

Novel mutations were highlighted in bold; VUS, variant of uncertain significance; NA, not applicable.

### Relationship between MSI status and germline mutations

**Figure 3:**
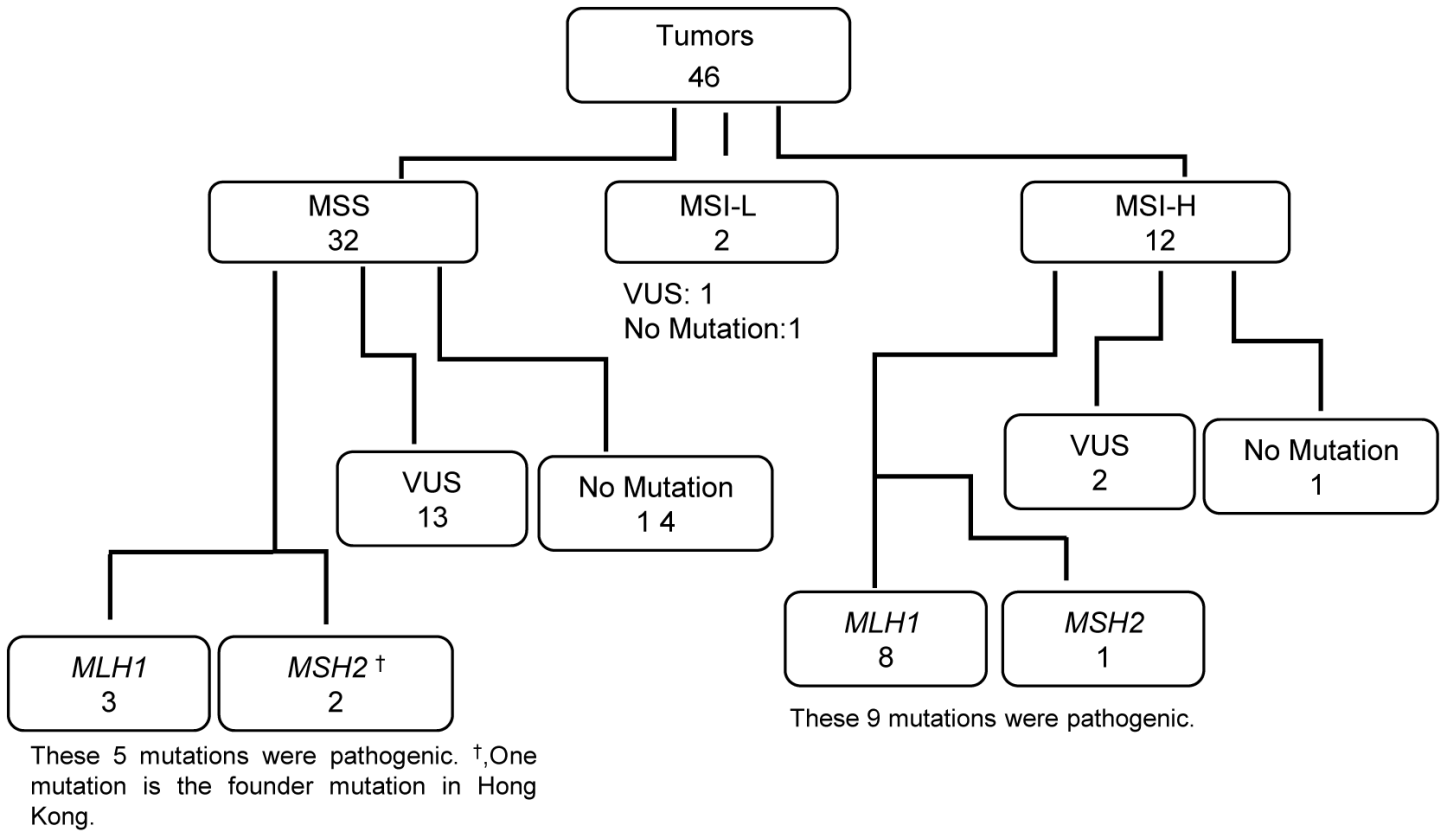
Correlation between tumor MSI status and germline mutations. Number in each box, case numeration. VUS, variant with uncertain significance.

In 46 CRC patients from whom colorectal tissues were available, 12 CRC tumors (26.1%) were classified as MSI-H, two tumors (4.3%) as MSI-L, and remaining 32 tumors (69.6%) as MSS (Figure 3). Among 14 LS patients with pathogenic mutations identified, 9 were identified with MSI-H tumors, demonstrating 64.3% sensitivity by MSI test. In patients with MSS tumours, 18 out of 32 (56.2%) patients were found to have germline mutations and five of these 18 patients (27.8%) harbored pathogenic mutations.

### Genotypic-phenotypic correlation

We further examined the relation between pathogenic germline mutations identified against typical phenotypic features of LS in Western populations (Table 3). Age at onset of initial tumor or first CRC was inversely associated with presence of pathogenic germline mutations. Mean age at onset of initial tumor (CRC or extracolonic tumor) was 40.1 years and 50.3 years in pathogenic and non-pathogenic mutation carriers, respectively (*P*<0.005, Table 3). For the first CRC, it was 40.2 years and 51.3 years in pathogenic and non-pathogenic mutation carriers, respectively (*P*<0.005, Table 3). In our series, we once again noted that there were predominantly left sided CRCs (*N* =  44 vs. 21). Right-sided CRCs (11 out of 21, 52.4%) were however more often associated with pathogenic mutations as compared with left-sided tumors (7 out of 44, 15.9%) (*P*  = 0.004, Table 3). There was however no significant association with tumor differentiation, histological subtype, presence of extracolonic malignancies, synchronous or metachronous CRCs (*P* >0.05 for all, Table 3). Interestingly, pathogenic germline mutations were significantly associated with earlier stage CRC (e.g., Dukes' A or B) as compared to non-pathogenic mutation carriers (62.5% vs. 37.5%, *P*  = 0.017, Somers' D test). Advanced tumor stages were however significantly associated with left-sided CRCs (data not shown).

**Table 3:** Evaluation of cardinal clinico-pathological features of Lynch Syndromes (LS) as predictors of pathogenic germline mutations.

	No. of patients with pathogenic mutation (%)	Remarks
**Clinical diagnostic criteria**		
Amsterdam-I (*N* = 40)	11 (27.5%)	NS (Somers' D test)
Amsterdam-II (*N* = 36)	10 (27.8%)	
Japanese Criteria (*N* = 2)	2 (100.0%)	
Mean age at onset (SD) of initial tumor		
40.1 (9.7) years vs. 50.3 (13.9) years in pathogenic mutation carriers (*N* = 23) and others (*N* = 48), respectively		*P*<0.005 (t-test with Bonferroni correction)
**Mean age at onset (SD) of CRC**		
40.2 years (10.0) vs. 51.3 (13.9) years in pathogenic mutation carriers (*N* = 20) and others (*N* = 45), respectively		*P*<0.005 (t-test with Bonferroni correction)
**Location of CRC lesion** †		
Right-sided (*N* = 21)	11 (52.4%)	*P* = 0.004 (Somers' D test)
Left-sided (*N* = 44)	7 (15.9%)	
Multiplicity of cancer		
Synchronous CRC (*N* = 3)	0	NS (Fisher's test)
Non-synchronous CRC (*N* = 62)	20 (32.3%)	
Metachronous CRC (*N* = 4)	3 (75.0%)	NS (Fisher's test)
Non-metachronous CRC (*N* = 61)	17 (27.9%)	
Synchronous and/or metachronous cancer (*N* = 11)	5 (45.5%)	NS (Fisher's test)
Non-synchronous/metachronous cancer (*N* = 60)	18 (30.0%)	
**Extracolonic malignancy**		
Presence (*N* = 8)	3 (37.5%)	NS (Fisher's test)
Absence (*N* = 63)	18 (28.6%)	
**Histological features of CRC lesion**		
Mucoid/signet ring cell (*N* = 6)	2 (33.3%)	NS (Fisher's test)
Adenocarcinomatous (*N* = 48)	12 (25.0%)	
Poorly differentiated (*N* = 6)	4 (66.7%)	NS (Somers' D test)
Moderately-differentiated (*N* = 47)	11 (23.4%)	
Well-differentiated (*N* = 1)	0 (0%)	

† , CRC lesions include those from patients whose initial tumors were extracolonic malignancies.

NS, not significant, *P* >0.05.

In our series, 21 patients from 19 families (19/59, 32.2%) did not exhibit any germline defect. When compared to 23 LS cases with pathogenic mutations identified, these 21 patients had significantly later onset of initial tumor or first CRC, predominance of left-sided CRC, higher incidence of advanced lesions (Dukes C or D) and MSS tumors (*P* <0.05 in all tests).

## Discussion

To date, this is the most comprehensive mutation profile of suspected LS families predominantly defined by Amsterdam criteria, in South-East Asia. In these 59 families, five common MMR genes were investigated. Pathogenic mutations were however confined to *MLH1* and *MSH2* and accounted for 60% (9/15) and 40% (6/15) of all identifiable mutations respectively. The predominance of *MLH1* and *MSH2* MMR mutation results are similarly reported in other ethnic databases [2]. It is important however to point out that our results differ from another local study [20] in which 10 out of 11 deleterious mutations (90.9%) were found in *MLH1* and remaining one in *MSH2* (9.1%). One of the main reasons we hypothesise is a differing selection criteria as the majority of cases (76 out of 78, 97.4%) in our cohort were Amsterdam-defined. In contrast, only 14% of participants in Lee SC et al [20] fulfilled Amsterdam criteria. It is our opinion that our results may in fact be a more precise reflection of our local LS kindreds.

In our study cohort of Singaporean LS families, each family was noted to harbour distinct deleterious germline mutations once again highlighting the high molecular heterogeneity of Lynch syndrome (Table 1). Interestingly, we have also found a high proportion of novel variants (6 pathogenic, 20 VUS and 2 SNPs), which make up 46.7% (28/60) of all variants identified in this cohort. This may suggest, that our South-East Asian ethnic groups in Singapore (primarily the Chinese and the Malay), thus have distinct mutational variants from Western populations. These results underscore the need to gain comprehensive mutational profiles for our local LS kindreds, which can facilitate appropriate screening and diagnostic algorithms in our population and neighbouring countries where similar ethnic groups reside. No doubt, further functional studies on those novel mutations are warranted.

Despite the high degree of heterogeneity observed, one recurrent mutation (*MLH1* c.793C>T) was identified in five patients from three unrelated Chinese families, accounting for 21.7% (5/23) of LS cases. This mutation, located at the beginning of exon 10, would lead to skipping of exon 10. In Taiwanese Chinese, founder effect of this mutation has been established based on 13 LS families [22]. While some of the Chinese families may have similar ethnic roots as the Taiwanese cohort, it is premature to conclude that *MLH1* c.793C>T is also a founder mutation in Singapore due to the small sample size. Nonetheless, our finding may lead to the establishment of cost-effective LS screening protocol for Singaporean Chinese. This mutation hotspot could be examined prior to exhaustive mutation scanning of common MMR genes. In addition, as migration worldwide has become increasingly easy, screening of this hotspot may be useful in various other regions if patients in similar ethnic backgrounds present with suspected LS. Certainly, additional studies are warranted to clarify the possible founder effect of this mutation in the local Chinese LS kindreds.

There are several noteworthy features observed in our cohort. We report a large deletion encompassing the first six exons of *MSH2* in one Chinese family. Analysis of the proband's tumor revealed lost expression of MSH2 protein. This is the first description of a large deletion in Singapore and it accounts for 5.9% (1 out of 17) of local LS families. This prevalence is slightly lower than other reports in various ethnic groups ranging from 6- 20% [23], especially in North America where this has been described as a founder mutation [24]. Although promoter methylation was examined in seven DNA repair genes, no families in this cohort were identified positively. These results suggest that the determination of large genomic rearrangement and promoter methylation of MMR genes should not be of top priority in the molecular screening in the local context.

In current clinical practice, selection of suspected LS cases for genetic testing rely on family history of cancers, clinico-pathological features typical of LS, and laboratory screening tests of tumor tissues (MSI and IHC). In this Singaporean cohort predominantly defined by Amsterdam criteria, pathogenic mutations were found in 29.5% (23 out of 78) of the study cohort (Table 1). This is lower than average detection rates in the Western populations [25]. In our series, heterozygous *BRAF* V600E mutation was identified in two tumors (MSS and MSI-H each, data not shown) and the corresponding two probands exhibited wild type alleles in germline DNA, indicating somatic mutation events. Comprehensive MMR gene scanning in these two families identified two VUS that, predicted *in silico*, are most likely of no deleterious impact on MMR functions. Given that tumor *BRAF* V600E mutation has been widely advocated as a negative predictor for LS-associated CRCs [26]–[30], there is a high likelihood that these two cases are likely sporadic CRCs or arise from hypermethylation pathways. While these two cases may be confidently excluded as LS patients, it may also be argued that the rest of the study cohort (19 families) with no germline defects, containing features such as a later age of onset of CRC, predominance of left-sided location of tumours, advanced Duke stage and a higher association with MSS status, are similar to that of sporadic CRCs. They may also potentially be classified as familial colorectal cancer type X. What is apparent however is that pathogenic mutation data on Asian LS populations remain inadequate compared with traditional Caucasian databases. We will also have to await confirming studies in similar ethnic groups, before these novel mutations identified in our series, may be confidently defined as LS pathogenic mutations in future. It is evident at this current juncture, that selection of LS families for genetic testing utilising Amsterdam Criteria in Asian families may be grossly inadequate with current data available.

It is well-known that not all pathogenic germline mutations causing functional impairment would influence the antigenicity of a MMR protein and result in diminished IHC staining. The 92.9% sensitivity for indicating pathogenic mutations by IHC are thus within expectation. Our results are in similar agreement to published studies [31]. Given relatively high concordance achieved between IHC and germline mutations, its convenience and cost effectiveness, IHC screening of tumors can be considered as a screening tool in Singapore and neighbouring South-East Asian countries for LS families, pinpointing “target” MMR genes most eligible for downstream mutation evaluation.

MSI-H is a hallmark feature associated with Lynch Syndrome. Despite the utility of mononucleotide markers, which has greater sensitivity than di-nucleotide markers for MMR mutations prediction, the detection rate of 26.1% (12 out of 46 tumors) for MSI-H is much lower compared to Western populations [32], [33]. Noteworthy is in our series, over half (18 out of 32, 56.2%) of our patients with MSS tumors had germline mutations, in which 27.8% (5 out of 18) were pathogenic mutations, including a founder mutation (*MSH2*, c.1457-1460delAATG) in Hong Kong [34]. It is known that inappropriate tumor dissection could retain adjacent normal colorectal cells into tumor specimens thus resulting in false negative MSI (i.e., MSS) results. In our study, a trained scientist had carefully dissected all tumor tissues prior to DNA extraction and at least 85% of cells analysed were cancerous cells, making technical errors under control. There are also multiple inherent limitations of MSI testing as current clinical practise reviews only a handful of markers out of more than one million microsatellite markers in the human genome [35]. Our results thus show that patients with MSS tumors in current clinical practise may be erroneously excluded from further mutation scanning for MMR defects. We are thus cautious utilising MSI as a screening mechanism for LS families in view of its suboptimal results and question whether the consensus marker panel recommended in the revised Bethesda guidelines is adequate for South-East Asian populations. IHC in our population may thus be superior to MSI as a screening tool for deficient MMR.

In conclusion, we report the most comprehensive evaluation of South-East Asian LS suspected families using current clinical criteria. In this cohort predominantly defined by Amsterdam criteria, all pathogenic germline defects appear to only involve *MLH1* and *MSH2*. The mutational spectrum is highly heterogeneous with mutations (including VUS) scattered throughout all five MMR genes investigated. There are also multiple novel variants that have never been previously described. Caution should be applied to overreliance on the anamnestic Amsterdam I or II criteria for case definitions in South-East Asian LS families. The utility of microsatellite marker panel recommended in the revised Bethesda guidelines may also not be suitable in Asian populations. Nonetheless, despite the limitations of our study, our results may provide an initial guide to a molecular diagnostic algorithm. We advocate to start with pre-screening in tumors by IHC and the germline hotspot mutation testing at *MLH1* c.793C>T. This is followed by thorough mutation scanning of small alterations in *MLH1* and *MSH2* and subsequent evaluation for large genomic rearrangement and epimutations, finally followed by mutation scanning of the remaining MMR genes. As molecular analyses in this study were performed in parallel rather than sequentially, the results reported here can be used to guide the development of efficient screening strategies.

## Supporting Information

Table S1 Amsterdam I and II criteria, Revised Bethesda guidelines and Japanese criteria for Lynch Syndrome.
(DOCX)

Table S2 Variants of uncertain significance (VUS) and polymorphisms in Singaporean Amsterdam-defined cohort.
(DOCX)
