# Supplementary material for: Systematic Study on Genetic and Epimutational Profile of a Cohort of Amsterdam Criteria-Defined Lynch Syndrome in Singapore
Source: PLoS One. 2014 Apr 7;9(4):e94170. doi: 10.1371/journal.pone.0094170 (PMC3978005; doi:10.1371/journal.pone.0094170)
Supplement: Table S1 — Amsterdam I and II criteria, Revised Bethesda guidelines and Japanese criteria for Lynch Syndrome. (DOCX) [file pone.0094170.s001.docx]

**Table S1**. Amsterdam I and II criteria, Revised Bethesda guidelines and Japanese criteria for Lynch Syndrome

| **Amsterdam I criteria (families must fulfill all criteria)** |
| --- |
| 1. At least 3 relatives should have histologically verified colorectal cancer (CRC); 1 of them should be a first-degree relative to the other 2. |
| 2. At least 2 successive generations should be affected. |
| 3. In 1 of the relatives, CRC should be diagnosed under 50 years of age. |
| 4. Familial adenomatous polyposis (FAP) should be excluded. |
| **Amsterdam II criteria (families must fulfill all criteria)** |
| 1. There should be at least 3 relatives with an HNPCC-associated cancer (colorectal, endometrium, stomach, small bowel, ureter or renal pelvis, brain, hepatobiliary tract and skin). |
| 2. One should be a first-degree relative of the other 2. |
| 3. At least 2 successive generations should be affected. |
| 4. At least one of the syndrome-associated tumors should be diagnosed before age 50. |
| 5. FAP should be excluded in the colorectal case(s), if any. |
| 6. Tumors should be verified by pathological examination. |
| **Revised Bethesda Guidelines for tumor testing for MSI (fulfillment of any one of the following is sufficient)** |
| 1. Colorectal cancer diagnosed in a patient who is less than 50 years of age. |
| 2. Presence of synchronous or metachronous colorectal or HNPCC associated tumors. |
| 3. Colorectal cancer with the Microsatellite instability-high (MSI-H) histology diagnosed in a patient who is less than 60 years of age.(Presence of tumor infiltrating lymphocytes, Crohn disease-like lymphocytic reaction, mucinous/signet-ring differentiation or medullary growth pattern) |
| 4. Colorectal cancer or HNPCC-associated tumor diagnosed under age 50 years in at least one first-degree relative. |
| 5. Colorectal cancer or HNPCC-associated tumor diagnosed at any age in two first-or second-degree relatives. |
| **Japanese criteria (fulfillment of any one of the following sets is sufficient)**  1. Three or more colorectal cancers among the first-degree relatives. |
| 2.Two or more colorectal cancers among the first-degree relatives and with any of the following: |
| a. Age at onset of colorectal cancer(s) less than 50 years old. |
| b.Right colon involvement. |
| c.Synchronous or metachronous multiple colorectal cancers. |
| d.Extracolorectal malignancy. |
