# Supplementary material for: Systematic Study on Genetic and Epimutational Profile of a Cohort of Amsterdam Criteria-Defined Lynch Syndrome in Singapore
Source: PLoS One. 2014 Apr 7;9(4):e94170. doi: 10.1371/journal.pone.0094170 (PMC3978005; doi:10.1371/journal.pone.0094170)
Supplement: Table S2 — Variants of uncertain significance (VUS) and polymorphisms in Singaporean Amsterdam-defined cohort. (DOCX) [file pone.0094170.s002.docx]

**Table S2.** Variants of uncertain significance (VUS) and polymorphisms in Singaporean Amsterdam-defined cohort

| **No. of Family** | **No. of subject** | **Nucleotide change and effect on protein** | **Nature of variant** | **LOVD** ^†^  (Consensus InSiGHT Classification Criteria) | **HGMD** ^‡^ |
| --- | --- | --- | --- | --- | --- |
| 1 | 2 | *MLH1*/Intron 13, c.1558+14G>A | VUS | Class 1, Not pathogenic; reported 34 times | Probably dis causing ^#^ |
| 1 | 1 | *MLH1*/Exon 18, c.2042C>T, p.Ala681Val | VUS | Class 3, Uncertain, reported twice | Dis causing |
| 1 | 1 | *MLH1*/Exon 18, c.2101C>A, p. Gln701Lys | VUS | Class 2, Likely not pathogenic; reported 8 times. Out of 4 functional assays or *in silico* analysis, 1:2:1 as pathogenic:non-pathogenic:inconclusive | Probably dis causing |
| 1 | 1 | MSH2/Exon 1 c.23C>T, p. Thr8Met | VUS ^§^ | Class 1, Not pathogenic | Dis causing |
| 1 | 3 | *MSH2*/Exon 3, c.505A>G, p. Ile169Val | VUS | Class 2, Likely not pathogenic; reported 13 times. Out of 6 *in silico* analysis, 2:3:1 as pathogenic:non-pathogenic:inconclusive |  |
| 4 | 5 | *MSH2*/Exon 7, c.1168C>T, p.Leu390Phe | VUS | Class 1, Not pathogenic; reported 38 times. Out of 10 functional assays or *in silico* analyses, 3:6:1 as pathogenic:non-pathogenic:inconclusive | Probably dis causing |
| 1 | 2 | *MSH2*/Exon 11, c.1690A>G, p. Thr564Ala | VUS | Class 1, Not pathogenic; reported 5 times | Conflicting information |
| 4 | 6 (Mutant); 3 (W/T) * | *MSH2*/Exon 12, c.1886A>G, p Gln629Arg | VUS | Class 1, Not pathogenic; reported before | Likely non- pathogenic |
| 1 | 1 | ***MLH1*/Exon1, c.101-102delAGinsTT, p.Glu34Val** | **Novel VUS** | No record | No record |
| 1 | 1 | ***MLH1*/Intron 1, c.117-10G>A** | **Novel VUS** | No record | No record |
| 1 | 2 | ***MLH1*/Exon 4, c.320T>A, p.Ile107Lys** | **Novel VUS** | No record | No record |
| 1 | 1 | ***MLH1*/Intron 7, c. 589 -46 G>C.** | **Novel VUS** | No record | No record |
| 1 | 2 | ***MSH2*/Intron 2, c.366+88G>A** | **Novel VUS** | No record | No record |
| 1 | 1 | ***MSH2*/Intron 11, c.1761 -12 A>G** | **Novel VUS** | No record | No record |
| 1 | 2 | **MSH2/Exon 5, c.809T>C p. Leu270Pro**. | **Novel VUS** | No record | No record |
| 1 | 1 | **MSH2/Exon 14, c.2425G>A, p. Glu809Lys**. | **Novel VUS** | No record | No record |
| 1 | 1 | ***MSH6*/Exon 3, c.464A>C, p.Lys155Thr** | **Novel VUS** | No record | No record |
| 1 | 1 | ***MSH6*/Exon 4, c.2246G>C, p.Gly749Ala** | **Novel VUS** | No record | No record |
| 1 | 1 | ***MSH6*/Exon 4, c.2531T>C, p.Met844Thr** | **Novel VUS** | No record | No record |
| 1 | 3 | ***MSH6*/Exon 5, c.3227G>A, p.Arg1076His** | **Novel VUS** | No record | No record |
| 1 | 1 | ***PMS2*/Intron 2, c.164-108 G>C** | **Novel VUS** | No record | No record |
| 1 | 1 | ***PMS2*/Exon 7, c.718 A>T, p.Ile240Phe** | **Novel VUS** | No record | No record |
| 1 | 4 | ***PMS2*/Exon 11, c.1928 A>G, p.Gln643Arg** | **Novel VUS** | No record | No record |
| 3 | 3 | ***PMS1*/promoter, -6 nucleotide from start of Exon1, G>T** | **Novel VUS** | No record | No record |
| 1 | 1 | ***PMS1*/Exon 1, c.-441G>T** | **Novel VUS** | No record | No record |
| 1 | 1 | ***PMS1*/Exon 1, c.-103-102insGGGTGC** | **Novel VUS** | No record | No record |
| 1 | 1 | ***PMS1*/Intron 7, c.812-128C>T** | **Novel VUS** | No record | No record |
| 1 | 1 | ***PMS1*/Intron 13, c.2991+20 A>G** | **Novel VUS** | No record | No record |
| 1 | 1 | *MLH1*/Exon 8, c.655A>G, p.Ile219Val | Disease-associated SNP ^$^ | Class 1, Not pathogenic; MAF ^¥^ >1%; reported 227 times. Out of 35 functional assays or *in silico* analysis, 23:12 as non-pathogenic:inconclusive | Probably disease-associated polymorphism |
| 2 | 2 | *MLH1/*Exon 12, c.1151T>A, p.Val384Asp | SNP ^[S1]^ | Class 1, Not pathogenic; MAF>1%; reported 43 times. Out of 11 functional assays or *in silico* analyses, 4:5:2 as pathogenic:non-pathogenic:inconclusive | Probably dis causing mutation |
| 2 | 5 | *MSH2*/Intron 9, c.1511 -91G>T | SNP | Class 1, Not pathogenic; MAF>1%; reported twice | No record |
| 4 | 8 | *MSH2*/Intron 10, c.1661+12G>A. | SNP | Class 1, Not pathogenic; MAF>1%; reported 78 times | Probably dis causing mutation |
| 2 | 2 | *MSH2*/Intron 12, c.2006 -6 T>C | Disease-associated SNP | Class 1, Not pathogenic; MAF>1%; reported 90 times | Disease associated polymorphism |
| 1 | 1 | *MSH6*/Exon 6, c.3488A>T, p.Glu1163Val | SNP | Class 2, Likely not pathogenic, MAF=0.018 in Chinese population. | Probably dis causing mutation |
| 2 | 2 | *MSH6*/Intron 8, c.3801+54C>G | SNP | MAF>1% in Chinese populations | No record |
| 3 | 3 | *MSH6*/Exon 10, c.4068-4071 (insTTGA at 4070), p.Lys1358AspfsX2 | Functional SNP | Class 2, Likely not pathogenic; MAF=0.035 | Probably *in vitro* or *in vivo* functional polymorphism |
| 1 | 1 | *PMS2*/Exon 5, c.379 G>A, p.Ala127Thr | SNP annotated in dsSNP database | No record | No record |
| 3 | 3 | *PMS2*/Exon 11, c.1454 C>A, p.Thr485Lys | SNP annotated in dsSNP database | Reported 7 times | No record |
| 4 | 4 | *PMS2*/Exon 11, c.1532, C>T, p.The511Met | SNP annotated in dsSNP database | Reported once | No record |
| 1 | 1 | *PMS2*/Exon 11, c.1621 G>A, p Glu541Lys | SNP | Class 1, not pathogenic, MAF>1%. | No record |
| 9 | 12 | *PMS2*/Intron 11, c.2006 +6 G>A | SNP | Class 1, not pathogenic, MAF>1%. | No record |
| 2 | 2 | *PMS2*/Exon 15, c.2570 G>C, p. Gly857Ala | SNP | Class 1, not pathogenic, MAF>1%. | No record |
| 1 | 1 | *PMS1*/Exon 13, c.2755C>T, p.Arg919Cys | SNP annotated in dsSNP database | No record | No record |
| 2 | 2 | ***PMS2*/Intron 9, c.989-162G>A** | **Novel SNP**  **(based on our series)** | No record | No record |
| 3 | 3 | ***PMS2*/Intron 9, c.989-256A>T** | **Novel SNP**  **(based on our series)** | No record | No record |

^†^, LOVD, London open variation database (V2.0 build 35) maintained by International Society of Gastroenterological Hereditary Tumors (InSiGHT). Variants were classified according to Consensus InSiGHT Classification v.1.9: 5/09/2013.

^‡^, HGMD, human gene mutation database. Dis causing, disease causative mutation; Our data were checked against this database as of 10 Jan 2014.

^#^, Probably dis causing, probably disease-causative;

*, W/T, wild type;

^$^, SNP, single nucleotide polymorphism

^§^, This missense mutation is classified as disease causing according to the HGMD classification criteria, but “Not pathogenic” according to the Consensus InSiGHT Classification criteria.

^¥^, MAF, minor allele frequency

Putative novel mutations were highlighted in bold.

^[S1]^, Fan Y, Wang W, Zhu M, Zhou J, Peng J, et al. (2007) Analysis of hMLH1 missense mutations in East Asian patients with suspected hereditary nonpolyposis colorectal cancer. Clin Cancer Res 13: 7515-7521.
